# Trends and variations in breast and colorectal cancer incidence from 1995 to 2011: A comparative study between Texas Cancer Registry and National Cancer Institute’s Surveillance, Epidemiology and End Results data

**DOI:** 10.3892/ijo.2015.2881

**Published:** 2015-02-06

**Authors:** ZHEYU LIU, YEFEI ZHANG, LUISA FRANZIN, JANICE N. CORMIER, WENYAW CHAN, HUA XU, XIANGLIN L. DU

**Affiliations:** 1 Department of Epidemiology, Human Genetics, and Environmental Sciences, University of Texas Health Science Center, Houston, TX, USA; 2 Department of Biostatistics, University of Texas Health Science Center, Houston, TX, USA; 3 Department of Management, Policy and Community Health, School of Public Health, University of Texas Health Science Center, Houston, TX, USA; 4 The University of Texas M.D. Anderson Cancer Center, Houston, TX, USA; 5 The University of Texas School of Biomedical Informatics, Houston, TX, USA

**Keywords:** breast cancer, colorectal cancer, incidence rates, trends, relative risks

## Abstract

Few studies have examined the cancer incidence trends in the state of Texas, and no study has ever been conducted to compare the temporal trends of breast and colorectal cancer incidence in Texas with those of the National Cancer Institute’s Surveillance, Epidemiology and End Results (SEER) in the United States. This study aimed to conduct a parallel comparison between the Texas Cancer Registry and the National Cancer Institute’s SEER on cancer incidence from 1995 to 2011. A total of 951,899 breast and colorectal cancer patients were included. Age-adjusted breast cancer incidence was 134.74 per 100,000 in Texas and 131.78 per 100,000 in SEER in 1995–2011, whereas age-adjusted colorectal cancer incidence was 50.52 per 100,000 in Texas and 49.44 per 100,000 in SEER. Breast cancer incidence increased from 1995 to 2001, decreased from 2002 to 2006, and then remained relatively stable from 2007 to 2011. For colorectal cancer, the incidence increased in 1995–1997, and then decreased continuously from 1998 to 2011 in Texas and SEER areas. Incidence rates and relative risks by age, gender and ethnicity were identical between Texas and SEER.

## Introduction

Breast cancer is the most commonly diagnosed cancer in women in the United States, while colorectal cancer is one of the top three most commonly diagnosed cancers among men and women (1–3). It is estimated that there will be 136,830 new colorectal cancer cases and 235,030 new breast cancer cases in 2014 in the USA (4–6). The incidence rates for breast and colorectal cancer have been declining consistently in recent years among all age groups and ethnicities (7–9). The decreasing incidence trends in breast and colorectal cancer have been attributed to earlier detection and more advanced treatment (10–14). Although cancer incidence rates have declined since the early 1990’s, the burden of cancer and its complications remains high and has a significant impact on human health (15–17).

A number of studies have examined Surveillance, Epidemiology and End Results (SEER) data for national cancer incidence trends and variations by age, ethnicity, cancer stage and other factors in the USA (18–23). However, the SEER data do not include Texas, a large and diverse state. The Texas Cancer Registry collects data on cancer in Texas. Few studies have examined the cancer incidence trends in the state of Texas (24–26), and no study has ever been conducted to compare the temporal trends of breast and colorectal cancer incidence in Texas with those of SEER. Therefore, in this study, we used the National Cancer Institute’s SEER data and Texas Cancer Registry (TCR) data to examine whether the overall incidence trends for both breast and colorectal cancer have similar patterns in the TCR and in SEER areas over the past 17 years from 1995 to 2011 (2,27). We also examined the variations in cancer incidence rates by age, gender, ethnicity, tumor stage and tumor grade in the TCR and SEER areas. The findings from this parallel comparison are expected to provide a significant overview of cancer incidence trends at the state and national level, and also to identify important factors associated with a decreasing risk of cancer, which are critical to enhance cancer prevention and control.

## Materials and methods

### Data sources

The SEER (Surveillance, Epidemiology and End Results) public-use dataset and the TCR (Texas Cancer Registry) limited-use dataset were utilized for this study (2,27). The SEER program, supported by the National Cancer Institute, includes population-based tumor registries in selected geographic areas in the USA. Because of our study comparison between the TCR and SEER for cancer cases in 1995 through 2011, we selected the following 9 SEER areas: Atlanta, Connecticut, Detroit, Hawaii, Iowa, New Mexico, San Francisco-Oakland, Seattle-Puget Sound and Utah, accounting for ~9.4% of the USA population (2). By the year of 2010, there were 18 SEER registries that covered 26.2% of the USA population (2). Because our study aimed to compare the incidence trends in breast and colorectal cancer from 1995 to 2011, only these nine registries, which had complete records on tumor stage and tumor grade in the study period, were included. The SEER registries ascertain all newly diagnosed (incident) cancer cases from multiple reporting sources such as hospitals, outpatient clinics, laboratories, private medical practitioners, nursing/convalescent homes/hospices, autopsy reports and death certificates (2). The TCR is a statewide and population-based cancer registry and is Gold Certified by the North American Association of Central Cancer Registries (27). The TCR dataset was determined to cover at least 95% statewide data for all cancer cases diagnosed from 1995 through 2011 in Texas. The denominator of population data used to calculate incidence rates were acquired from the U.S. Census Bureau’s Population Estimates Program (28). The Committee for the Protection of Human Subjects at the University of Texas Health Science Center in Houston approved this study.

### Study population

We identified all women diagnosed with breast cancer and men and women diagnosed with colorectal cancer as their primary tumor in 1995–2011 from both SEER and TCR. In the 9 SEER registries, 328,142 patients with breast cancer and 224,511 patients with colorectal cancer were included. In Texas, 243,695 women with breast cancer and 155,551 patients with colorectal cancer were included.

### Study variables

The primary outcome of interest was the incidence rates of breast and colorectal cancer from 1995 to 2011. Incidence rates were defined as the number of new cases in one calendar year divided by the total population at risk in the same year (29). Breast cancer cases were identified using the ‘Primary Site’ variable in both SEER and TCR, coded as C500–C509 according to ‘International Classification of Diseases for Oncology, Third Edition (ICD-O-3), and Topography Section (2,7)’. Colorectal cancer cases were coded as C180–C189, C199, C209, and C260 (2). According to the methods by Wu *et al* in counting total colorectal cancer cases, colon included the cecum (C180), appendix (C181), ascending colon (C182), hepatic flexure (C183), transverse colon (C184), splenic flexure (C185), descending colon (C186), sigmoid colon (C187), and large intestine, NOS(C188–C189,C260) (30). The rectum included the rectosigmoid junction (C199) and the rectum-not otherwise specified (C209).

The independent variables of interest in this study included age, gender, ethnicity, tumor stage and tumor grade. Age was classified according to five categories with <50, 50–59, 60–69, 70–79 and ≥80 years. Gender was a binary variable with male and female, but for breast cancer, only women were included because of the rarity of diagnosis in men. Ethnicity was categorized as white, black and other. Tumor stage was classified as localized, regional, distant and unknown. Localized stage was confined within the breast and colon. Regional stage was defined as tumor involvement of the regional lymph nodes, primarily those in the axilla, to be involved. Distant stage was defined as the cancer metastatic to other parts of the body as well. The unknown stage was defined as having missing information of cancer status (31). Tumor grade represented the level of differentiation, in which poorly differentiated cancer cells usually divide more quickly and therefore represent more aggressive malignancies (32). Tumor grade at diagnosis was stratified into four categories: differentiated, moderately differentiated, poorly differentiated and undetermined.

### Statistical analysis

Annual incidence rates of breast and colorectal cancer cases per 100,000 persons were age-adjusted to the 2000 US standard population stratified by five age groups. The incidence rates were computed by age-group, gender, race, tumor stage, and tumor grade for breast and colorectal cancer separately. We used the SEER^*^Stat software to calculate incidence rates by dividing the number of new cancer cases in each category over the total population at risk (33). The SAS statistical software was used to calculate incidence rates with 95% confidence intervals for TCR data. Poisson regression model with population size specific to demographic groups as offset variable were used to determine the association between incidence rates and potential risk factors. Covariates included age-group, gender, race, tumor stage and tumor grade. In order to determine the temporal relationship, risk ratios between two cancer incidence rates were calculated and adjusted by potential confounders. The assumption of the Poisson model were examined by examining constant variance plots of the variables. No specific pattern was detected in the outputs indicating that the constant variance was valid.

## Results

Table I presents the total number of patients diagnosed with breast and colorectal cancer in Texas and in 9 SEER areas. From 1995 to 2011, there were 243,695 new breast cancer cases and 155,551 colorectal cancer cases in Texas, and 328,142 breast cancer cases and 224,511 colorectal cancer cases in 9 SEER areas. The mean age for breast cancer was 60.5 years in the TCR and 61.7 years in SEER, and the mean age for colorectal cancer was 67.2 years in the TCR and 69.2 years in SEER. The age distribution of these cancer cases was similar overall in the TCR and SEER, although the proportion of younger patients appeared to be slightly higher in Texas. Over 50% of new breast cancer cases occurred in those aged ≥60 years, while >70% of new colorectal cancer cases occurred in those aged ≥60 years. There were a slightly more male colorectal cancer cases in the TCR and SEER, and >80% of patients were white. The overall distribution by tumor stage and tumor grade was similar between Texas and SEER, but the proportion of cancer cases with unknown tumor stage and undetermined tumor grade was higher in the TCR than that in SEER.

**Figure 1:**
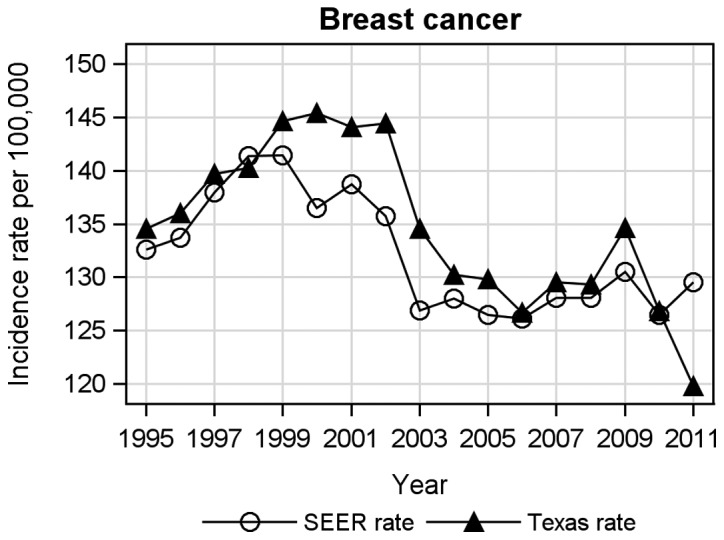
Breast cancer age-adjusted incidence rates over time by year (1995–2011).

**Figure 2:**
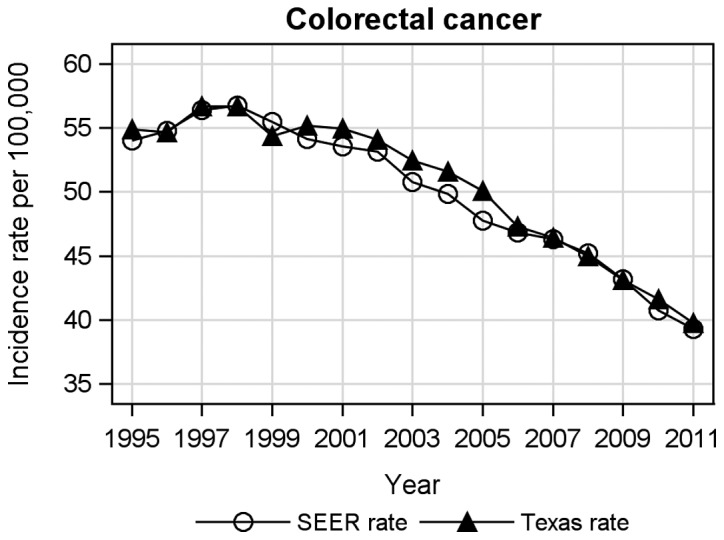
Colorectal cancer age-adjusted incidence rates over time by year (1995–2011).

Fig. 1 presents parallel comparisons of age-adjusted incidence trends from 1995 to 2011 for breast cancer between the TCR and SEER areas, whereas Fig. 2 presents the age-adjusted incidence trends from 1995 to 2011 for colorectal cancer in the TCR as compared to SEER. The overall incidence trends and changing patterns over the 17-year periods for breast and colorectal cancer were almost identical between the TCR and SEER areas. Specifically, breast cancer incidence increased from 1995 to 2001, decreased from 2002 to 2006, and then remained relatively stable from 2007 to 2011 (Fig. 1). The increased breast cancer incidence in 1995–2001 was consistent with the time period when the widespread use of screening program was implemented (34). For colorectal cancer, the incidence increased in the first three years between 1995 and 1997, and then decreased continuously from 1998 to 2011 in both Texas and SEER areas.

**Figure 3:**
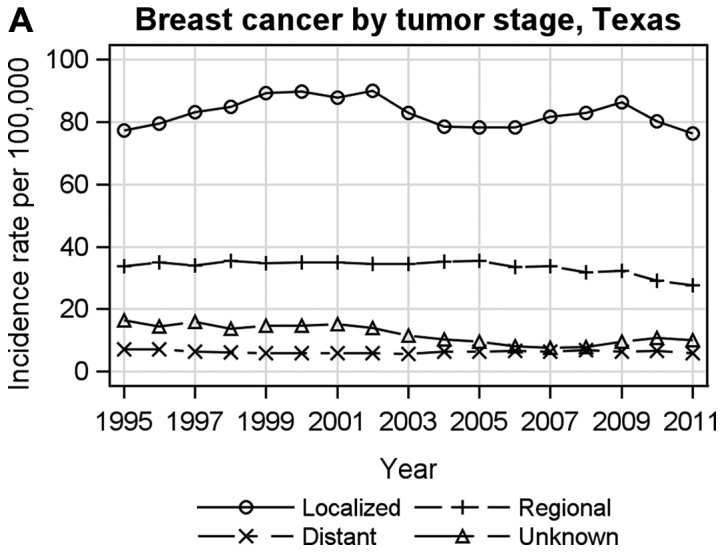

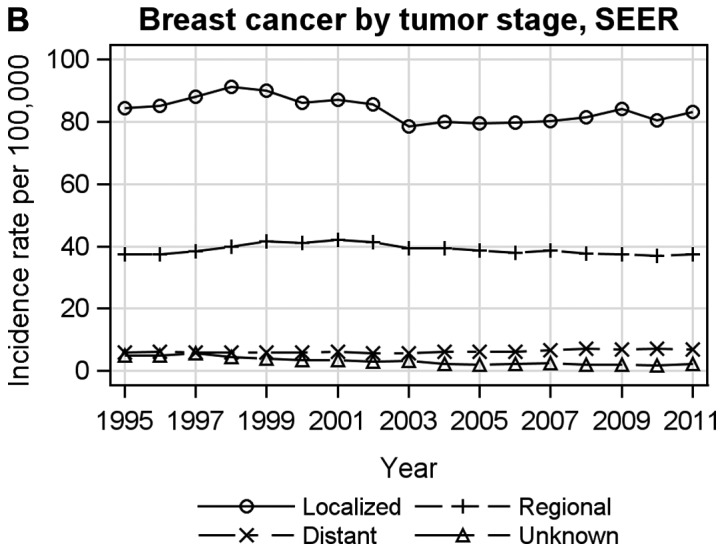
Breast cancer age-adjusted incidence rates over time by tumor stage, 1995–2011. (A) Texas. (B) SEER.

**Figure 4:**
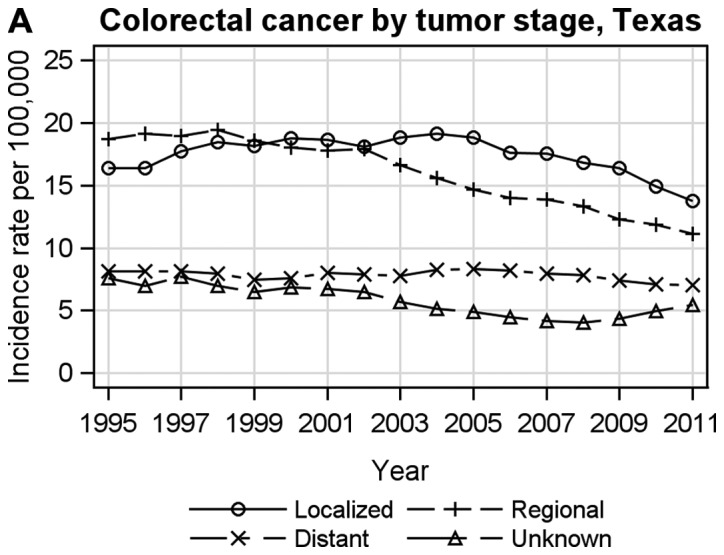

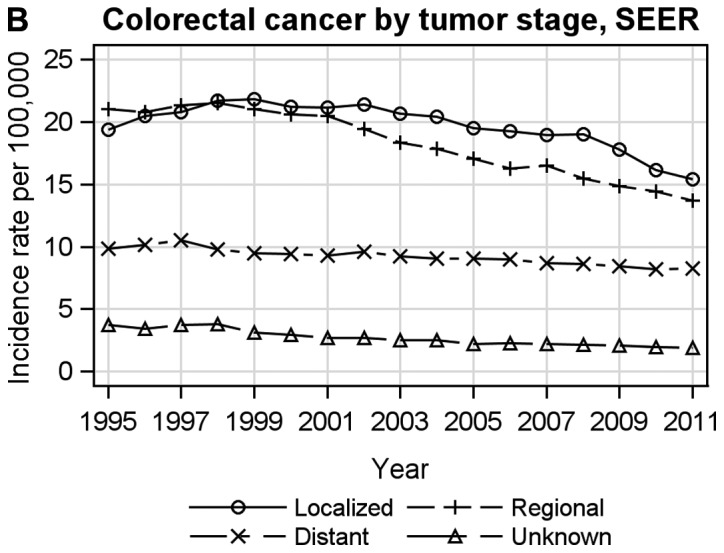
Colorectal cancer age-adjusted incidence rates over time by tumor stage, 1995–2011. (A) Texas. (B) SEER.

Figs. 3 and 4 present the age-adjusted tumor stage-specific incidence rates for breast and colorectal cancer in the TCR and SEER. For breast cancer, tumor stage-specific incidence rates were similar between the TCR (Fig. 3A) and SEER (Fig. 3B), in which incidence for localized breast cancer increased early on and then decreased, while the incidence for distant stage breast cancer was stable with a slight increase over time. For colorectal cancer, the incidence for all stages decreased over time in the TCR (Fig. 4A) and in SEER (Fig. 4B) except for an increase for unknown stage colorectal cancer in the TCR from 2008 to 2011.

Table II presents the age-adjusted incidence rates of breast cancer and the relative risks of cancer incidence by patient and tumor characteristics in the TCR and SEER. The overall age-adjusted breast cancer incidence in 1995–2011 was 134.74 per 100,000 in the TCR and 131.78 per 100,000 in SEER. The specific incidence rates and relative risks by age, gender and ethnicity were also similar between in the TCR and SEER after adjusting for tumor stage and grade. For example, as compared to those <50 years of age, women ≥60 were >9 times more likely to develop breast cancer, whereas American Indians and Asian-Pacific Islanders were significantly less likely to develop breast cancer and African Americans had a marginally lower risk of developing breast cancer as compared to whites.

Similarly, Table III presents the age-adjusted incidence rates of colorectal cancer and relative risks of cancer incidence stratified by patient characteristics in the TCR and SEER after adjusting for tumor stage and grade. Overall age adjusted colorectal cancer incidence rate from 1995 to 2011 was 50.52 per 100,000 in the TCR and 49.44 per 100,000 in SEER. Because the mean age for developing colorectal cancer was older than that for breast cancer, those aged 60–69 years were >23 times more likely to develop colorectal cancer and those aged ≥80 years were >58 times more likely to develop this disease than those <50 years of age. The risk of developing colorectal cancer was noted to be significantly lower in women than in men. African Americans had a higher risk of developing colorectal cancer as compared to whites.

## Discussion

This parallel comparison study between the TCR and SEER reported a number of significant findings. The overall incidence trends for both breast and colorectal cancer were noted to have similar patterns from 1995 to 2011. The breast and colorectal cancer incidence rates by age, gender, ethnicity, tumor stage and tumor grade in the TCR were also similar to those in SEER areas. These cancer incidence trends over time and variations by other factors are important findings when monitoring cancer progress, assessing the success of cancer prevention and control, and identifying high-risk populations for additional intervention. Furthermore, the identical cancer incidence trends reported in this comparative study can also be viewed as important evidence of the validity of the TCR’s incidence data. This is because the National Cancer Institute’s SEER has been established since 1973 and is often considered the gold standard in cancer registries (2).

This study demonstrated that breast cancer incidence increased from 1995 to 2001, decreased from 2002 to 2006, and then was relatively stable from 2007 to 2011. The interval of increased breast cancer incidence was consistent with the time period when widespread use of early detection for breast cancer such as screening mammography programs were implemented. According to a study by Swan *et al*, the increased use of mammography during the late 1990’s resulted in a dramatically increased number of breast cancer cases among females in the USA (35). Many other studies also supported this finding (36,37). After the peak time increase in 2001, breast cancer incidence continued to decline and became relatively stable over the past several years. A number of studies have suggested that the decreased incidence rates may be attributable to reduction in the use of peri-menopausal hormone therapy, decreases in utilization of mammography and decreases in the number of preclinical cases detected by screening in recent years (38–44).

Similarly, we found that colorectal cancer incidence increased from 1995 to 1997 and then continued to decrease from 1998 to 2011 in both the TCR and SEER areas. We did not observe any dramatically increasing trend period associated with colorectal cancer screening. Several previous studies have reported similar findings and conclusions (7,8,29). This may be related to the nature and gradual adoption of screening tools for colorectal cancer over time (13,14). The U.S. Preventive Service Task Force has recommended that fecal occult blood testing and sigmoidoscopy be used for colorectal cancer screening since the 1990s for persons aged ≥50 (45). The federal Medicare program began covering the cost of colonoscopy screening for colorectal cancer since 2001 for individuals with an average-risk of developing colorectal cancer (46). Colorectal cancer is known to occur later in life with a mean age at 70 years (68 for men and 72 for women) (47,48). We found that men had a higher risk of developing colorectal cancer than women in both the TCR and SEER. Our finding is consistent with the study by Cook *et al* who concluded that males had much higher risk (RR, 1.36) for colorectal cancer than females from 1995 to 2004 (49). Differences in colorectal cancer incidence rates stratified by ethnicity can largely be explained by differences in education level, smoking status and health insurance status (50–55). In our analysis, the black population had highest colorectal cancer incidence rates. Possible explanations include a larger percentage of the smoking population in blacks; the highest prevalence of cigarette smoking is also known to occur among individuals with high school or lower education (56–58).

Although TCR and SEER datasets are known to be comprehensive and of high quality, a number of factors may have affected the findings. First, we were unable to verify specific populations by year in each registry, which might have resulted in biased calculations for the annual incidence rates. Second, we studied the 9 SEER areas that accounted for ~9% of the USA population, and therefore the results may not be generalizable to all SEER areas or to the entire USA population. Furthermore, a number of important known risk factors for breast and colorectal cancer such as smoking, family history, physical exercise and environmental factors are not included in these datasets and cannot be studied. The differences in these factors may have affected the cancer incidence comparisons.

In conclusion, breast and colorectal cancer incidence trends from 1995 to 2011 were almost identical between the TCR and SEER areas. Breast cancer incidence increased in 1995–2001 and decreased afterwards, while colorectal cancer incidence decreased continuously from 1998 to 2011. Older age was a significant risk factor for the high risk of developing cancer, particularly for colorectal cancer. The cancer risk also varied according to gender and race/ethnicity. Additional studies may be needed to explore smaller geographical areas within these registries and environmental factors associated with the changing incidence trends.

## Figures and Tables

**Table I:** Number (proportion) of patients with breast and CRC in the Texas Cancer Registry (TCR) and SEER, 1995–2011, stratified by patient and tumor factors.

	No. (%) of cancer cases			
	
Patient and tumor characteristics	TCR (%)	SEER (%)	TCR (%)	SEER (%)
Mean age	60.46	61.73	67.15	69.16
Age (years)				
<50	60017 (24.63)	74066 (22.57)	17359 (11.16)	20190 (8.99)
50–59	59481 (24.41)	76676 (23.37)	28088 (18.06)	35423 (15.78)
60–69	55660 (22.84)	72686 (22.15)	37406 (24.05)	48532 (21.62)
70–79	43744 (17.95)	62866 (19.16)	40976 (26.34)	62987 (28.06)
≥80	24793 (10.17)	41848 (12.75)	31722 (20.39)	57379 (25.56)
Gender				
Male			82571 (53.08)	113388 (50.50)
Female	243695 (100)	328142 (100)	72980 (46.92)	111123 (49.50)
Race/ethnicity				
White	210903 (86.54)	269958 (82.65)	132793 (85.37)	181261 (81.14)
Black	26161 (10.74)	30525 (9.35)	19428 (12.49)	22513 (10.08)
Other (American Indian/Asian/Pacific Islander)	6631 (2.72)	26135 (8.00)	3330 (2.14)	19609 (8.78)
Tumor stage				
Localized	149999 (61.55)	207998 (63.39)	57800 (37.16)	88825 (39.56)
Regional	61325 (25.16)	96063 (29.27)	52374 (33.67)	81824 (36.45)
Distant	11567 (4.75)	15808 (4.82)	26594 (17.10)	41776 (18.61)
Unknown	20804 (8.54)	8273 (2.52)	18783 (12.08)	12086 (5.38)
Tumor grade				
I: Well differentiated	34324 (14.08)	63902 (19.47)	11562 (7.43)	18962 (8.45)
II: Moderately differentiated	77260 (31.70)	123943 (37.77)	84543 (54.35)	128657 (57.31)
III: Poorly differentiated	75475 (30.97)	100962 (30.77)	23173 (14.90)	36499 (16.26)
IV: Undetermined	56636 (23.24)	39335 (11.99)	36273 (23.32)	40393 (17.99)
Total	243695	328142	155551	224511

**Table II:** Age-adjusted incidence rates of breast cancer in the Texas Cancer Registry (TCR) and in SEER, 1995–2011.

	Breast cancer in TCR		Breast cancer in SEER	
		
Characteristics	Incidence (95% CI)^a^	RR (95% CI)^b^	Incidence (95% CI)^a^	RR (95% CI)^b^
Age (years)				
<50	43.03 (42.24–43.82)	1.00 (Reference)	44.17 (43.85–44.49)	1.00 (Reference)
50–59	287.02 (274.65–299.38)	6.58 (6.50–6.65)	267.75 (265.86–269.66)	6.16 (6.10–6.23)
60–69	405.00 (392.57–417.44)	9.34 (9.23–9.45)	394.61 (391.75–397.50)	9.29 (9.20–9.39)
70–79	461.24 (444.70–477.78)	10.63 (10.50–10.76)	463.28 (459.66–466.92)	11.74 (11.62–11.87)
≥80	404.07 (389.27–418.87)	9.27 (9.14–9.41)	419.42 (415.40–423.46)	12.06 (11.91–12.20)
Race/ethnicity				
White	136.14 (131.91–140.37)	1.00	136.45 (135.93–136.97)	1.00
Black	131.90 (129.40–134.40)	0.98 (0.97–0.99)	123.33 (121.93–124.75)	0.93 (0.92–0.94)
Other (American Indian/Asian/Pacific Islander)	98.51 (89.74–107.27)	0.77 (0.75–0.79)	96.75 (95.58–97.94)	0.72 (0.71–0.73)
Total	134.74 (130.88–138.60)		131.78 (131.32–132.23)	

a Incidence rate was number of cases per 100,000 population and was age adjusted to the 2000 US population (28).

b Incidence ratio (relative risk) was adjusted for age, race, tumor stage and tumor grade.

**Table III:** Age-adjusted incidence rates of CRC in the Texas Cancer Registry (TCR) and in SEER, 1995–2011.

	Colorectal cancer in TCR		Colorectal cancer in SEER	
		
Characteristics	Incidence (95% CI)^a^	RR (95% CI)^b^	Incidence (95% CI)^a^	RR (95% CI)^b^
Age (years)				
<50	6.11 (5.92–6.30)	1.00 (Reference)	6.03 (5.95–6.12)	1.00 (Reference)
50–59	68.46 (67.00–69.92)	11.29 (11.07–11.50)	63.13 (62.47–63.79)	10.60 (10.42–10.79)
60–69	145.38 (137.06–153.69)	23.76 (23.33–24.19)	139.91 (138.67–141.16)	23.37 (22.98–23.76)
70–79	245.37 (225.95–264.79)	41.05 (40.33–41.79)	262.84 (260.79–264.90)	45.09 (44.38–45.82)
≥80	342.33 (314.44–370.23)	58.68 (57.60–59.78)	371.32 (368.29–374.37)	65.51 (64.46–66.59)
Gender				
Male	60.78 (57.26–64.29)	1.00	57.49 (57.15–57.83)	1.00
Female	42.70 (40.18–45.21)	0.70 (0.69–0.71)	43.12 (42.87–43.38)	0.75 (0.74–0.76)
Race/ethnicity				
White	49.37 (46.52–52.22)	1.00	48.87 (48.65–49.10)	1.00
Black	64.84 (61.68–67.99)	1.35 (1.33–1.37)	58.43 (57.65–59.22)	1.21 (1.19–1.22)
Other (American Indian/Asian/Pacific Islander)	36.69 (33.71–39.67)	0.75 (0.72–0.77)	42.67 (42.07–43.27)	0.88 (0.86–0.89)
Total	50.52 (47.67–53.38)		49.44 (49.23–49.64)	

a Incidence rate was number of cases per 100,000 population and was age adjusted to the 2000 US population (28).

b Incidence ratio (relative risk) was adjusted for age, gender, race, tumor stage and tumor grade.
